# Transitioning to adult mental health services for young people with ADHD: an Italian-based survey on practices for pediatric and adult services

**DOI:** 10.1186/s13034-023-00678-9

**Published:** 2023-11-28

**Authors:** Elisa Roberti, Francesca Scarpellini, Rita Campi, Michele Giardino, Antonio Clavenna, Maurizio Bonati, the TransiDEA group, Patrizia Stoppa, Ottaviano Martinelli, Paola Morosini, Giuseppe Capovilla, Maria Antonella Costantino, Federico Raviglione, Patrizia Conti, Giorgio Rossi, Maria Teresa Giarelli, Elisa Maria Fazzi, Marialuisa Carpanelli, Maria Paola Canevini, Francesco Rinaldi, Massimo Molteni, Aglaia Vignoli, Renato Borgatti, Laura Farinotti, Donatella Arcangeli, Paola Bona, Franco Giovannoni, Maurizio Pincherle, Roberto Canitano, Elena Gennaro, Chiara Caucci, Carmela Bravaccio, Stefano Sotgiu, Giancarlo Costanza, Eleonora Briatore, Benedetto Vitiello, Giuseppe Zappulla, Elisa Colombi, Mariarosa Ferrario, Malida Franzoi, Federica Martinez, Laure Obino, Marco Carrozzi, Federico Durbano, Marco Grignani, Marco Grignani, Alessandro Antonucci, Angelo Rella, Pietro Di Paolo, Germano Fiore, Gianluca Piemontese, Giulio De Nicola, Andres Conca, Angelo Cucciniello, Moro Anna Rosa, Giorgio Francobandiera, Maria Carla Moraca, Paola Corsini, Alessandro Antonucci, Emi Bondi, Luisa Aroasio, Giuseppe Imperadore, Daniela Malagamba, Camilla Callegari, Marco Toscano, Gialuigi Di Cesare, Corrado Cappa, Francesco Gardellin, Elio Laudani, Nicoletta Raschitelli, Michele Zanetti, Massimo Cartabia, Vanna Graziani, Federico Marchetti, Tosca Suprani, Paolo Di Bartolo, Ilaria Viganò, Ilaria Costantino, Valeria Tessarollo, Giampaolo Ruffoni

**Affiliations:** 1 Laboratory of Epidemiology of Developing Age, Department of Medical Epidemiology, Istituto Di Ricerche Farmacologiche Mario Negri IRCCS, Via Mario Negri 2, 20156 Milan, Italy; 2 Information Science for Clinical Knowledge Sharing Unit, Department of Medical Epidemiology, Istituto Di Ricerche Farmacologiche Mario Negri IRCCS, Milan, Italy

**Keywords:** Transition, ADHD, Adult ADHD, Survey, Health services

## Abstract

**Background:**

Supporting young ADHD patients in transition to adult services is essential. Yet, the low percentages of successful referrals and the issues reported by patients and clinicians stress the need for further attention to transitioning practices. The present study assessed the transitioning process of Attention-Deficit/Hyperactivity Disorder (ADHD) patients in Child and Adolescent Mental Health Services (CAMHS) and Adult Mental Health Services (AMHS) in the Italian territory. We asked child and adult psychiatrists to report the current state of services and their observations on limitations and possible future matters that must be addressed.

**Method:**

Seventy-seven centers (42 CAMHS, 35 AMHS) filled in a web-based survey in which they reported the number of ADHD patients, how many transitioning patients they had within the past year, and how they structured transition.

**Results:**

A fragmented picture emerged from the survey. Lack of resources, training, and communication between services hinder the transition process, and many adult patients remain under CAMHS’ care. While some services have a protocol, there is no structured guidance that can help improve integration and continuity of treatment.

**Conclusion:**

The observed situation reflects a need for improvement and standard guidelines to enable a successful transition process, considering clinicians' and patients’ necessities.

## Background

Attention-Deficit/Hyperactivity Disorder (ADHD) is a common psychiatric disorder in childhood and adolescence, With an average prevalence of 5.9% based on symptoms criteria [1, 2] and In Italy, the prevalence is of 2.9% [3]. Diagnoses are generally made at primary school age (6–12 years) at child neuropsychiatry services. From the age of 6, methylphenidate (i.e., a central nervous system stimulant that contributes to hyperactivity and impulse control, commonly used to treat ADHD) can also be prescribed. Referral is made to ADHD centers of reference or a Child Neuropsychiatry. In some regions, specific diagnostic and therapeutic paths have been shared between centers (e.g., Lombardy) [4]. Still, there are no common national guidelines other than the ones provided by the SINPIA (Italian Society of Child Neuropsychiatry), mainly related to the drugs’ regulation. While ADHD is considered a neurodevelopmental syndrome that impacts children’s daily life functioning [5], it affects adults as well, with a prevalence of around 2.2% [1]. Compared with childhood ADHD, adult symptomatology lacks validated diagnostic criteria and is relatively neglected in epidemiological studies [6–8]. Notably, while most symptoms that have their onset in the pediatric age continue to manifest throughout adolescence, they often persist (albeit with less impact) in adult life for up to two-thirds of the patients [9]. Yet, adolescents in care for ADHD often stop treatment altogether, and adults with ADHD frequently fall out of the care net when transitioning to adult age. Sometimes this happens because these individuals disengage with the care system, although they might resort to it again if their symptoms aggravate or after a life crisis [10]. Other common reasons why young people stop accessing treatment after transitioning to adult age are: increased perception of medications’ adverse consequences, the idea of ADHD as a childhood disorder that does not require treatment for adults, changes in life circumstances leading to unintentional treatment cessation, lack of information and challenges in accessing services [11, 12]. The difficulties in accessing services are particularly relevant as should be addressed, since care may not be guaranteed even for patients that require it the most.

Supporting the transition phase is essential, as made evident by the low percentages (around 9%) of ADHD patients referred to adult services in some European countries [13, 14]. A review that included eight studies (six from the UK, one from Hong Kong, and one from Italy) analyzed the commonalities in perceived transition issues [15]. The emerging themes were summarized in five categories. The **first category** comprised the lack of information, preparation, transition planning, parallel care of the services, and families’ involvement. This domain also opened the question of the right transitioning age, with significant variations within and between studies. The **second category** was related to the lack of appropriate adult services and difficulties in identifying available services. The accessibility (i.e., long waiting lists and stigma associated with accessing a mental health service) and the high rates of unaccepted cases by adult services were also reported. The **third category** comprised the limited resources and competencies of adult services (i.e., need for more education, training, skill development, and adequate treatment individuation in clinicians of adult services). The **fourth category** described the inadequate care when pediatric clinicians held onto the patients beyond age boundaries or, conversely, the lack of supervision, leading to feelings of abandonment and perception of poor management of the condition. The **fifth category** considered the negative impact of a bad transitioning process on the patient's well-being.

In attempting to overcome these issues, standard protocols and guidelines were designed in a few countries. In the UK, the National Institute for Clinical Excellence (NICE 2008, 2019) other than providing criteria for diagnosing and managing ADHD in children suggested a shared planning to organize the transition process [16]. One of the recommendations enclosed in these guidelines is that services “*put in place systems of communication and protocols for information sharing among paediatric, child and adolescent, forensic, and adult mental health services for people with ADHD, including arrangements for transition between child and adult services*.” Similar protocols exist in other countries, although their efficacy is not ensured. For instance, the TRACK study in England [17, 18] and the IMPACT review in Northern Ireland [19] reported that very few patients receive optimal care in the transition process. The MILESTONE project [20] was the first to implement a new intervention applied in eight European countries to transitioning patients with several clinical diagnoses. The project showed that in the case of general mental disorders, a structured assessment of transition needs facilitates an appropriate planning process (shared between professionals, patients, and families). Nonetheless, this intervention was never specifically applied with neurodevelopmental disorders.

More work is needed to inform clinicians and families on how the ADHD transitioning process can be successful and how to overcome the difficulties due to the so-called ‘*leaky pipelines’* [21] through which patients disperse. Within the Italian National Health Service, to date there are no specific indications for and how to organize the transition to adult care. Just as there are no specific indications from the various scientific societies.

With the present study, we aim to fill this gap by considering the opinion of professionals working with transitioning ADHD patients in the Italian territory. In Italy, only two studies have assessed the situation, one at the single province level (i.e., Modena) [22], and one at the regional level for the Lombardy Region [23]. The latter involved a total of 18 services in an exploratory study investigating the health care management and continuity for young adults with ADHD. In this region, regional ADHD territorial services are accredited in regional hospitals, and therefore linked to CAMHS, and often deal with the transition process of ADHD patients towards a shift in care by AMHS. Nonetheless, what emerged from Reale and colleagues is that in 2018, out of these 18 centers, only four reported having a protocol to manage the transition process, and none of those protocols were specific for ADHD patients [23]. Moreover, while it seems that previously hospitalized adolescents have a 50% chance of being successfully transferred to an AMHS and that higher engagement was witnessed within the first 2 years [22], this rate was limited to a narrow territory (i.e., the Province of Modena). By collecting information on the available services, we assess the status of the ADHD transition process in the whole Italian territory. Moreover, by asking clinicians to report their observations on the transition process, we seek to evaluate whether their reported limitations are consistent with the five points highlighted by Price and colleagues (2019). Ideally, the collected evidence will be able to draw a picture of the current state of the procedures and design desirable future trajectories.

## Methods

The TransiDEA (Transitioning in Diabetes, Epilepsy and ADHD patients) is an Italian project that aims to define and evaluate the feasibility of treatment programs for the transition from adolescence to adulthood in the ADHD, Epilepsy, and Diabetes pathological fields. It involves patients, parents, and clinicians as part of a collaborative and shared project. As a first step, the aim was to define the current situation concerning how adult and pediatric services manage the transition phase. The present work reports findings limited to the ADHD centers. The head of 89 national Child and Adolescent Mental Health Services (CAMHS) and 257 Adult Mental Health Services (AMHS) received a letter explaining the project’s aims and the invitation to participate by completing the web-based questionnaire. It was also stated that the results would be reported and that they would be informed of the project's continuation. Three further reminders were sent to improve the recruitment rates. The survey was completed between December 2021 and May 2022.

Two surveys were developed, one for CAMHS and one for AMHS. The two versions differed in questions about sending (CAMHS) or receiving (AMHS) patients. The questions asked are similar to those asked in previous surveys [23, 24], shared with the other participants in the TransiDEA study group and the clinicians of a few centers. Based on their feedback, some minor changes were made (e.g., unclear terms or where a free text option was necessary). The final structure of the surveys was as follows:

- An initial information screening on the centers: region, type of service, number of ADHD patients, presence of a specific service dedicated to ADHD patients, and pharmacological treatments administered;
- Questions on the transition process (i.e., the process of preparing, planning and moving from children’s to adult services): number of patients in transition within the past year, sent to or coming from which services, for the services that formalize a preparation phase, starting from which age;
- Questions for centers that actively refer patients (i.e., formal request from one health professional to another to take on a patient, providing clinical information about them):

- Centers that refer to adult services (only for pediatric services): effective age at referral, criteria for referral (age, clinical characteristics, other aspects);
- Whether respondents feel that they received appropriate/sufficient information (only for adult services);
- Methods of the referral or receiving, of the patients (e-mail, telephone, letters, information sheets, or other forms), the time required, whether an interview is also planned (with the patient, with the family, or between the services), professional figures involved (psychiatrists, psychologists, nurses, administrative officers, therapists, social workers, educators);

- Whether it is beneficial that both services have an active role in the transition phase, how long they believe that an optimal transition should be, in how many sessions, whether both specialists’ teams should be present, and where it should be carried out (CAMHS, AMHS, a third facility, remotely, or if it does not matter);
- Whether services have a protocol (i.e., a set of predetermined evidence-based criteria regulating interventions for effective patient care management, guiding in particular how and when transition should be guaranteed in an appropriate care pathway) and whether it is specific for ADHD or generic;
- Whether services foresee a monitoring phase;
- Whether services also have disability care pathways;
- Questions on limitations, unmet needs, and desired changes in dealing with transitioning ADHD patients.

The study was notified to the IRCCS “Carlo Besta” Foundation Ethics Committee (8 September 2021, protocol n. 87). The study was performed in accordance with the ethical standards of the 1964 Declaration of Helsinki and its later amendments. We followed the Strengthening the Reporting of Observational Studies in Epidemiology (STROBE) reporting guidelines. The analyses reported are descriptive. Data are reported as the number and percentage of responders, and continuous variables are summarized by reporting median values and interval ranges, calculated in Microsoft Excel.

## Results

Out of 89 the CAMHS and 257 AMHS contacted, 77 centers (22% of those 346 contacted initially) filled in the survey (47% CAMHS and 14% AMHS) between December 2021 and May 2022. Within the Italian National Health Service, the organizational structure and distribution of CAMHS and AMHS is based on the resident population by age, so although the response rate is low it is presumable that there are no marked differences between the responding and non-responding centers. A description of the services is reported in Table 1.

**Table 1:** Number and geographical distribution of the services that took part in the web-based survey

	CAMHS (N = 42)	AMHS (N = 35)
Area		
North	33	23
Center	4	7
South	5	5
Number of total ADHD patients		
Minors	7262	–
Adults	210 (3% of ADHD patients)	656 (min 2—max 250)
Services that currently have adult ADHD patients	17 (40%)	23 (66%)
Services with a dedicated ADHD unit	33 (79%)	10 (29%)

The table also reports the number of ADHD patients, the number of services that currently have ADHD patients, and the number of services that have a dedicated ADHD unit

### Services that referred patients

**Fig. 1:**
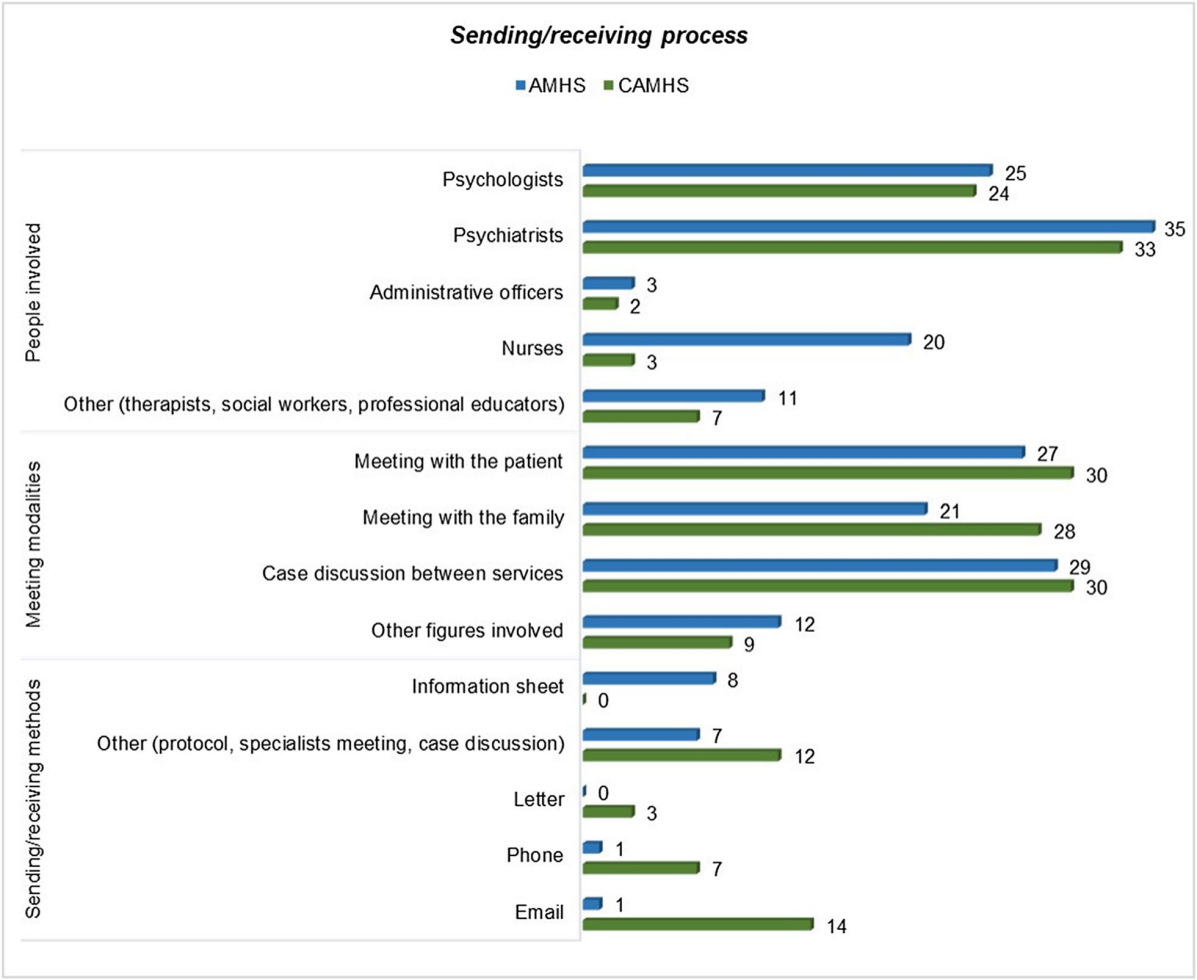
Methods, time required, interviews planned, and professional figures involved in the transition process for both CAMHS and AMHS. Compilers were given the opportunity to provide multiple responses

During the year preceding the survey, 36 CAMHS (85%) referred 15 to 21 year-old patients (Median = 18 years) to AMHS. Nonetheless, the desirable passage window would be narrower, ranging from a minimum of 17 years of age to a maximum of 19 years. The most frequent criteria that CAMHS used for patients’ selection for transitioning were age (83%) and clinical features (comorbidities, severe symptoms, and drug treatment) (31%). Eight of the 11 AMHS who received ADHD patients in transition received appropriate and sufficient information about the patients. Figure 1 summarizes the methods, the time required, the interviews planned, and the professional figures involved in the transition process. The main differences between child and adult services were that adult services reported involving nurses and other professional figures more than CAMHS, as well as the use of information sheets. CAMHS, on the other hand, favored different information and communication channels, such as e-mail, phone, and letters.

### The transition process

**Fig. 2:**
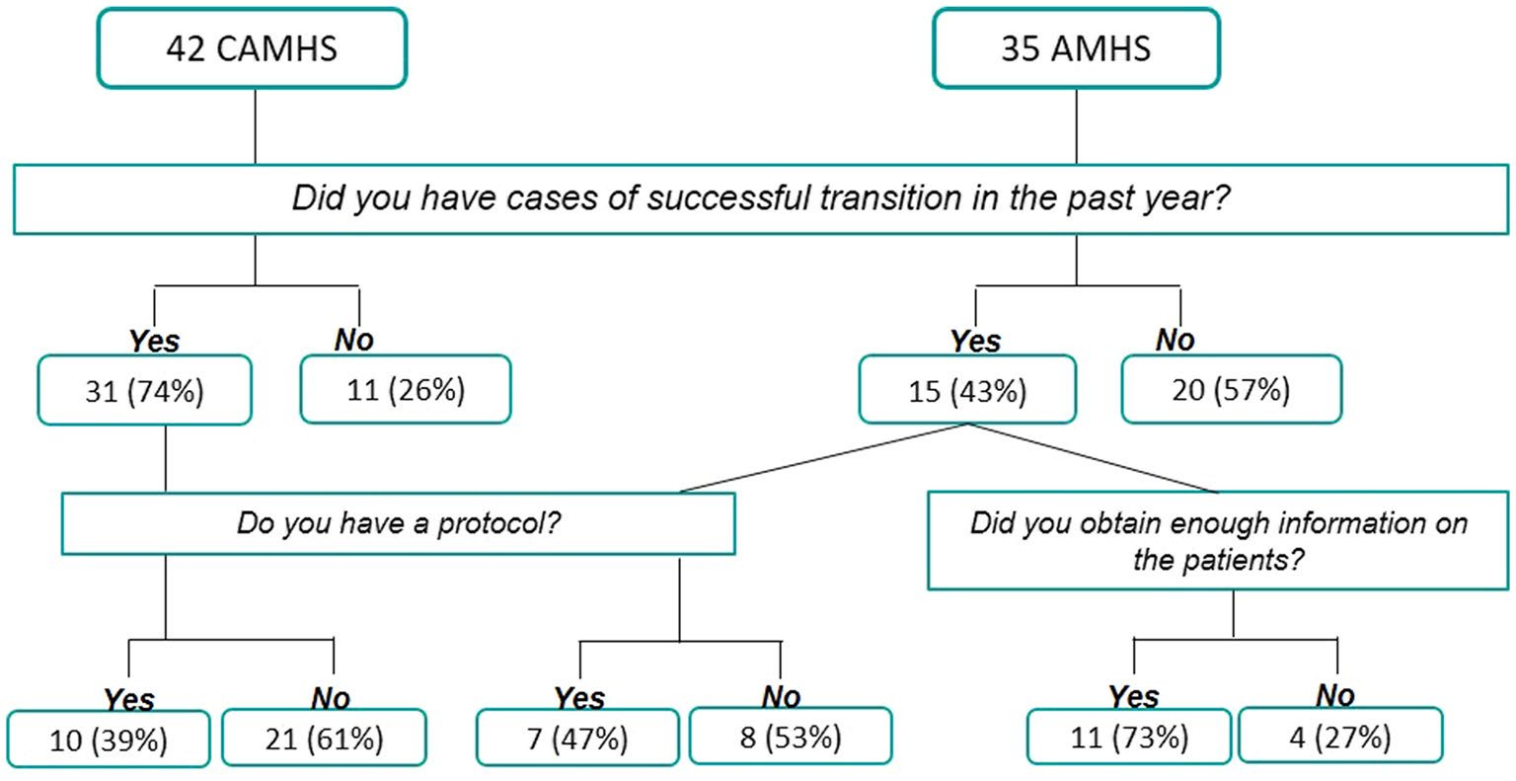
Numbers and percentages of the responses to the main questions of the survey

Seventy-four percent of the CAMHS (all with a dedicated service) reported cases of successful transition (31 of the 36 that referred patients), for a total of 206 patients, while 43% of the AMHS (8 with a dedicated team of specialists) had transitioning patients in the past year (n = 15), for a total of 167 patients. The majority of the CAMHS sent their patients to a private psychiatrist (80%, n = 34) while a smaller number to the AMHS (38%, n = 16). Most AMHS reported that their patients were coming from the CAMHS (40%, n = 14). Twenty-three CAMHS (55%) have a formalized preparation phase, ideally taking place at 17 years of age (range: 16–19). Eighty-three percent of the CAMHS (n = 35) prescribed methylphenidate and atomoxetine, while only 54.3% (n = 19) of the AMHS prescribed these drugs. The main results are summarized in Fig. 2.

### Services’ role in the transition

Sixty-nine percent of the CAMHS (n = 29) and 91% of the AMHS (n = 32) considered it beneficial that both services have an active role in the transition phase (11 of the AMHS and 22 of the CAMHS who received or sent ADHD patients in the previous year). CAMHS consider that an optimal transition should take two meetings (range: 1–6) in a nine-week window, while AMHS indicated seven meetings (range: 1–50). 15 of the CAMHS (36%) and 21 of the AMHS (91% of the 23 with ADHD patients) report that both specialists' teams should be present when discussing the case of a transitioning patient. Both types of services would prefer that meetings were carried out in their facilities (Table 2).

**Table 2:** CAMHS and AMHS responses to where meetings regarding transitioning ADHD patients should be carried out

	Where should the meetings with transitioning patients take place?	
	CAMHS (N = 15)	AMHS (N = 21)
CAMHS	7	4
AMHS	5	8
A third facility	0	2
Remotely	1	1
It does not matter	2	6

The total of 15 CAMHS and 21 AMHS refers to services that indicated that that both specialists' teams should be present when discussing the case of a transitioning patient

### Protocol, monitoring phase, and disability care pathways

Ten CAMHS (24% of all centers, 39% of those who sent patients in the previous year) and 11 AMHS reported having a transition protocol (32% of all centers, 50% of the centers with ADHD patients). Of these, only 3 CAMHS and 2 AMHS had a protocol specific to ADHD patients. Only 4 of the participating CAMHS (9%) and 5 of the AMHS (15%) reported foreseeing a monitoring phase. 22 of the CAMS (52%) and 5 of the AMHS (15%) plan disability care pathways.

### Limitations, unmet needs, and desired changes in dealing with transitioning ADHD patients

Twenty-seven CAMHS (64%) reported limitations, and 30 (71%) desired changes, while 19 AMHS (54%) reported limitations, 25 (71%) unmet needs, and 31 (89%) desired changes. Those who answered positively could write down what they perceived as a limitation or what they envisioned as a future possibility.

As for limitations, some emerging common themes pertained to the integration amongst services, communication issues, lack of resources, organizational problems, and lack of treatment continuity. CAMHS workers also described the lack of AMHS psychiatrists’ training as highly problematic, the lack of shared training, and the absence of reliable protocols. Moreover, AMHS reported the need to potentiate services, rehabilitation pathways, and ease-of-access to pharmacological treatments. The difficulty in dealing with diagnoses and disorders and issues in communicating with the families were also reported.

**Fig. 3:**
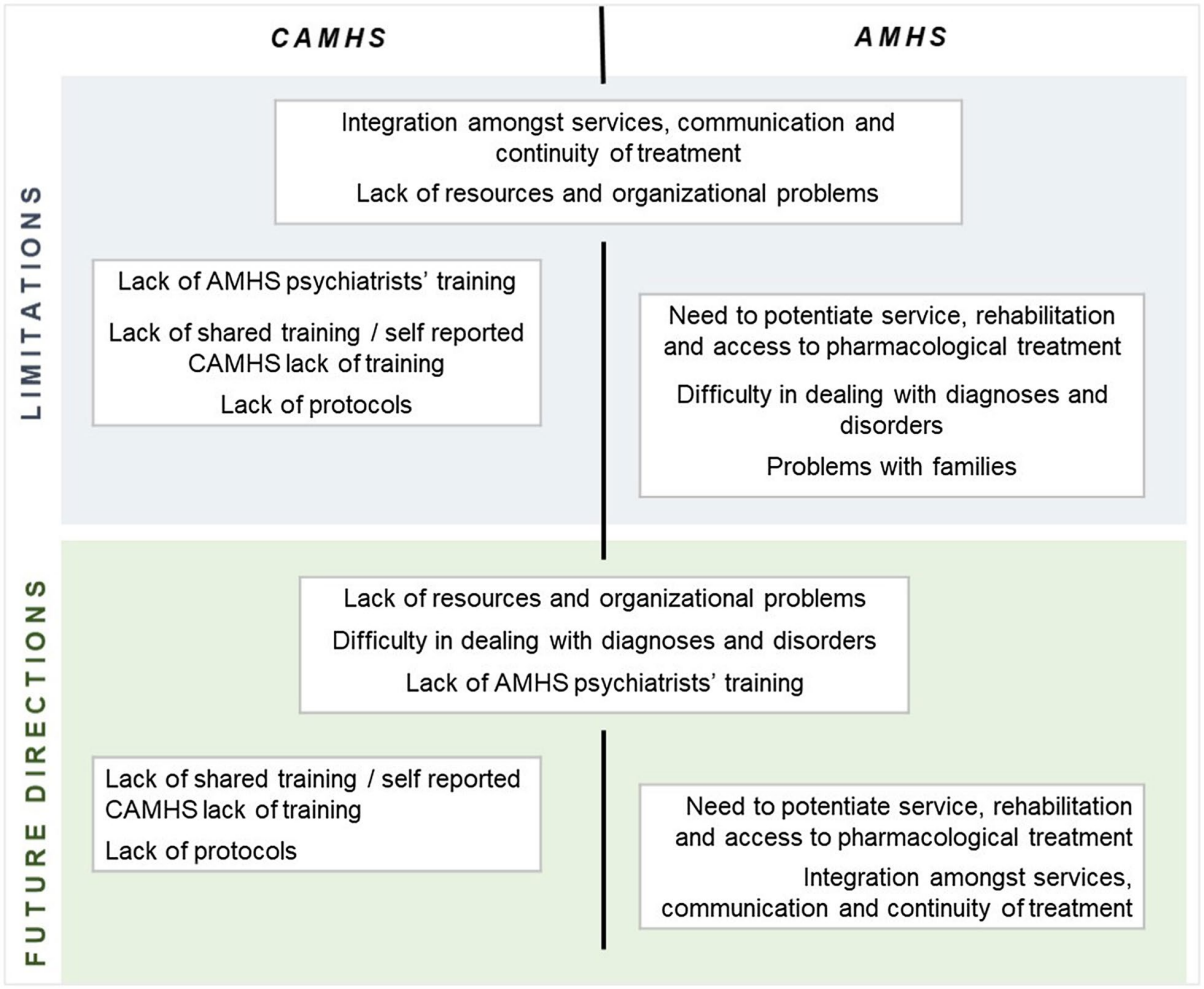
Limitations and desired changes in the future as reported by both CAMHS and AMHS. Some observations are shared (centered), others are limited to CAMHS (left side) or AMHS (right side)

Regarding future desired changes, both CAMHS and AMHS, again, reported working on the lack of resources and organizational problems, adding the implementation of strategies to better deal with the diagnostic process, and with the improvement of psychiatrists’ training**.** AMHS services added the need to work on services’ integration and communication, and CAMHS services listed the need to design standard protocols and shared training. AMHS centers did not foresee any functional changes in dealing with the families, although they perceived this as an issue. These themes are summarized in Fig. 3.

## Discussion

The transitioning process entails many changes and difficulties of which clinicians, patients, and families can be more or less aware. Although some guidelines exist in many countries (e.g., the NICE guidelines in the UK), their efficacy and generalizability have not yet been accurately evaluated. No Italian studies considered the professionals’ opinion, nor did they cover the whole national territory. Additionally, the factors contributing to a successful transition process in the country are unknown.

The present study aimed to draw a picture of the current state of the transitioning process in Italy. We asked the professional figures involved (i.e., CAMHS and AMHS workers) how the transitioning process happens and to describe the issues they perceive as problematic, along with their needs.

We achieved an acceptable geographical representation, with a total of seventy-seven respondents (42 working in CAMHS and 35 in AMHS) from 18 of the 20 Italian regions.

The analysis of the survey responses drew an interesting picture. While 74% of the CAMHS reported following transitioning patients in the previous year, only 43% of the AMHS stated the same. This means that many patients still get lost in the process. Some of these patients are sent to private psychiatrists or psychologists, falling out of the public health system nonetheless. In other cases, adult patients continue to be treated by CAMHS (in our sample, this happened for 210 patients, distributed in 40% of the CAMHS). Moreover, the ideal age range for transitioning would be between 17 and 19 years of age, while the time window actually needed is wider, involving patients between 15 and 21 years of age. Even when the transition is completed by the 18 years of age mark, the gap with what would be defined by protocols is considerable. On 7262 CAMHS patients, 206 transitioning patients were detected (around 3%). Indeed, this percentage is lower than reported elsewhere, at around 9% [14]. Still, appropriate information about the rate of transitioning ADHD patients, in particular in Italy, is scant [25]. A larger prevalence in transitioning patients is reported in cases of schizophrenia (56.6%), personality disorders (37.4%), and pervasive developmental disorders (autism) (32.4%) [26]. This dissimilarity is unsurprising given the greater impact on these conditions’ everyday lives. In comparison, ADHD patients may have lower percentages of transitioning, given the more negligible implications for day-to-day functioning and social interactions.

While these data suggest that organizational problems affect day-to-day transition process management, many more factors that hinder a successful transition are consciously reported as unmet needs or desired changes. Such observations largely overlap with the first three dimensions reported by Price and colleagues [15]. **First,** the lack of professionals' preparation, transition planning, protocols, and parallel care of the services. In this domain, we also included the absence of training and competencies in adult services. **Second,** the lack of appropriate adult services. The AMHS are aware of the need to potentiate the services they provide, such as rehabilitation pathways, and access to pharmacological treatment. **Third**, the limitations due to the lack of resources and organizational problems. Both CAMHS and AMHS professionals strongly felt this theme. Interestingly, what was highlighted in this domain is the need to develop strategies to efficiently follow the transition with the available resources rather than to increase the resources.

Only one AMHS raised the issue of communicating with the families. When describing how they arranged the meetings between services, only 21 AMHS and 28 CAMHS declared that they involve the patients’ families in the process. The families had, on the other hand, a central role in other studies [15, 27]. The scarce consideration of families in the Italian transition organization may contribute to the lack of a smooth passage, given that these patients are still vastly dependent on their parents’ support.

Moreover, a theme that often emerged was the lack of adequate training for professionals working in the AMHS. Once again, from the opposite viewpoint, more than half of those same professionals reported that they did not receive enough adequate information (only 48% said that they did). Another observation in this respect is that only 61% of the CAMHS that sent patients to AMHS in the previous year think it would be helpful to actively involve both services in the transition phase. Having formal guidelines to smooth the communication between the two parties in the process might also help improve communication and outcomes.

These observations shed attention on the need for specific protocols to address the needs of the ADHD population in the transition phase. We asked the 21 services that reported having a protocol whether they could send it to us to investigate whether guidelines already exist, need improvement, or if there is a complete lack of efficient protocols. Notably, while in 2018, in the Lombardy region [23], no pediatric centers were reported to have a specific protocol for one of the neurodevelopmental disorders, from our survey, it emerged that three pediatric centers do (2 within the same region). Nonetheless, they regard Autism Spectrum Disorder, Schizophrenia, and Behavioral Disorders, while no protocol specific to ADHD exists. Many more centers have a non-specific protocol (10 CAMHS and11 AMHS) or do not have a protocol (76% of CAMHS, 78% of AMHS). We received the full version of six protocols, four from CAMHS and two from AMHS. All were characterized by the working group’s definition, the number of meetings and the corresponding figures involved (i.e., patients, CAMHS, and AMHS specialists), and the criteria to select patients, including the age window. One protocol also included the possibility of defining personalized trajectories based on specific needs. With the available resources, defining individual care paths does not emerge as a priority, as confirmed by the fact that no respondent listed individual needs in future directions. Instead, it would be desirable to have flexible enough guidelines to allow for a certain degree of customization, considering the available assets. In our survey, only when provided space to write notes on the transition process did eight clinicians report more psychological considerations, such as the importance of paying attention to individuality, culture, and personal experiences concerning the care process. While most of these essential points were shared, the degree of detail differed widely. Suffice it to say that some protocols are only 2 to 5 pages long, while others are up to 25 pages long. In the future, the need for a standard protocol should be addressed. We believe that it should fall somewhere in between the existing ones: while short protocols are not detailed enough, excessively long protocols may be complicated to use daily in a reality of scarce resources.

A limitation of this study is the reduced sample size. Although the included services are not expected to differ from the ones that did not participate, reaching a wider number of professionals would have ensured a better representation on the practices adopted at a national level. Moreover, most of the services that took part in the survey are located in large cities, mostly in northern Italy. Nonetheless, we think that the merits of this survey’s observations are several. First, it constitutes the first attempt to draw a picture of the transitioning patients from the professionals’ point of view. It collects practical information and allows a highlighting of needs and desired changes. Moreover, it stands as the starting point for future work. While the descriptive data reported in this study are limited to an Italian based survey, its implications are not. Indeed, the main concerns and issues are the same in different countries (e.g., Italy, the UK and Hong Kong) [15]. The tools provided so far to regulate the transition process, such as the NICE guidelines (https://www.nice.org.uk/guidance/ng43) from the UK and the American Six Core Elements (https://www.gottransition.org/six-core-elements/) are very similar in principle. While cultural differences (between and within countries) cannot be overlooked, the effort towards designing new strategies can be shared and adapted internationally. As future steps of the TransiDEA project, the collected protocols will be analyzed in depth. Individual pathways of patients and their clinicians will then be analyzed, leading to the definition of standardized guidelines. To do so, individual stakeholders from across Italy, including patients, parents or caregivers, clinicians from CAMHS and AMHS, and possibly general practitioners [27] will be involved.

## Conclusions

A successful transition process is crucial for the future of ADHD patients. However, as reflected by this survey conducted in Italy, there are currently many difficulties hindering this process. The priority in the near future must be the definition of standard guidelines that can facilitate a successful transition.

## Data Availability

The data and the materials presented in this study are available on request from the corresponding author.

## Abbreviations

ADHD: Attention-Deficit/Hyperactivity Disorder
TransiDEA: Transitioning in Diabetes, Epilepsy and ADHD
CAMHS: Child and Adolescent Mental Health Services
AMHS: Adult Mental Health Services
NICE: National Institute for Clinical Excellence

## References

[1] Faraone SV, Banaschewski T, Coghill D, Zheng Y, Biederman J, Bellgrove MA (2021). The world federation of ADHD international consensus statement: 208 evidence-based conclusions about the disorder. Neurosci Biobehav Rev.

[2] Polanczyk GV, Willcutt EG, Salum GA, Kieling C, Rohde LA (2014). ADHD prevalence estimates across three decades: an updated systematic review and meta-regression analysis. Int J Epidemiol.

[3] Reale L, Bonati M (2018). ADHD prevalence estimates in Italian children and adolescents: a methodological issue. Ital J Pediatr.

[4] Bonati M, Scarpellini F, Cartabia M, Zanetti M, on behalf of the Lombardy ADHD Group (2021). Ten years (2011–2021) of the Italian Lombardy ADHD register for the diagnosis and treatment of children and adolescents with ADHD. Children.

[5] Reale L, Bartoli B, Cartabia M, Zanetti M, Costantino MA, Canevini MP (2017). Comorbidity prevalence and treatment outcome in children and adolescents with ADHD. Eur Child Adolesc Psychiatry.

[6] McGough JJ, Barkley RA (2004). Diagnostic controversies in adult attention deficit hyperactivity disorder. Am J Psychiatry.

[7] Sibley MH, Mitchell JT, Becker SP (2016). Method of adult diagnosis influences estimated persistence of childhood ADHD: a systematic review of longitudinal studies. Lancet Psychiatry.

[8] Song P, Zha M, Yang Q, Zhang Y, Li X, Rudan I (2021). The prevalence of adult attention-deficit hyperactivity disorder: a global systematic review and meta-analysis. J Glob Health.

[9] Faraone SV, Biederman J, Mick E (2006). The age-dependent decline of attention deficit hyperactivity disorder: a meta-analysis of follow-up studies. Psychol Med.

[10] Young S, Murphy CM, Coghill D (2011). Avoiding the “twilight zone”: recommendations for the transition of services from adolescence to adulthood for young people with ADHD. BMC Psychiatry.

[11] Titheradge D, Godfrey J, Eke H, Price A, Ford T, Janssens A (2022). Why young people stop taking their attention deficit hyperactivity disorder medication: a thematic analysis of interviews with young people. Child Care Health Dev.

[12] Hansen AS, Telléus GK, Mohr-Jensen C, Lauritsen MB (2021). Parent-perceived barriers to accessing services for their child’s mental health problems. Child Adolesc Psychiatry Ment Health.

[13] Kooij SJ, Bejerot S, Blackwell A, Caci H, Casas-Brugué M, Carpentier PJ (2010). European consensus statement on diagnosis and treatment of adult ADHD: the European network adult ADHD. BMC Psychiatry.

[14] Eke H, Ford T, Newlove-Delgado T, Price A, Young S, Ani C (2020). Transition between child and adult services for young people with attention-deficit hyperactivity disorder (ADHD): findings from a British national surveillance study. Br J Psychiatry.

[15] Price A, Janssens A, Woodley AL, Allwood M, Ford T (2019). Review: Experiences of healthcare transitions for young people with attention deficit hyperactivity disorder: a systematic review of qualitative research. Child Adolesc Ment Health.

[16] National Institute for Health & Care Excellence. NICE Clinical Guideline [CG177] Osteoarthritis: Care and Management in Adults National Institute for Health & Care Excellence: London 2019. 31869054

[17] Islam Z, Ford T, Kramer T, Paul M, Parsons H, Harley K (2016). Mind how you cross the gap! outcomes for young people who failed to make the transition from child to adult services: the TRACK study. BJPsych Bull.

[18] Singh SP, Paul M, Ford T, Kramer T, Weaver T, McLaren S (2010). Process, outcome and experience of transition from child to adult mental healthcare: multiperspective study. Br J Psychiatry.

[19] Leavey G, McGrellis S, Forbes T, Thampi A, Davidson G, Rosato M (2019). Improving mental health pathways and care for adolescents in transition to adult services (IMPACT): a retrospective case note review of social and clinical determinants of transition. Soc Psychiatry Psychiatr Epidemiol.

[20] Singh SP, Tuomainen H, Bouliotis G, Canaway A, De Girolamo G, Dieleman GC (2021). Effect of managed transition on mental health outcomes for young people at the child–adult mental health service boundary: a randomised clinical trial. Psychol Med.

[21] Anderson JK, Newlove-Delgado T, Ford TJ (2022). Annual research review: a systematic review of mental health services for emerging adults—moulding a precipice into a smooth passage. J Child Psychol Psychiatry.

[22] Pontoni G, Di Pietro E, Neri T, Mattei G, Longo F, Neviani V (2022). Factors associated with the transition of adolescent inpatients from an intensive residential ward to adult mental health services. Eur Child Adolesc Psychiatry.

[23] Reale L, Costantino MA, Sequi M, Bonati M (2018). Transition to adult mental health services for young people with ADHD. J Atten Disord.

[24] Reale L, Frassica S, Gollner A, Bonati M (2015). Transition to adult mental health services for young people with attention deficit hyperactivity disorder in Italy: parents’ and clinicians’ experiences. Postgrad Med.

[25] Ford T (2020). Transitional care for young adults with ADHD: transforming potential upheaval into smooth progression. Epidemiol Psychiatr Sci.

[26] Stagi P, Galeotti S, Mimmi S, Starace F, Castagnini AC (2015). Continuity of care from child and adolescent to adult mental health services: evidence from a regional survey in Northern Italy. Eur Child Adolesc Psychiatry.

[27] Price A, Mitchell S, Janssens A, Eke H, Ford T, Newlove-Delgado T (2022). In transition with attention deficit hyperactivity disorder (ADHD): children’s services clinicians’ perspectives on the role of information in healthcare transitions for young people with ADHD. BMC Psychiatry.

